# Response to Antenatal Cholecalciferol Supplementation Is Associated With Common Vitamin D–Related Genetic Variants

**DOI:** 10.1210/jc.2017-00682

**Published:** 2017-05-29

**Authors:** Rebecca J. Moon, Nicholas C. Harvey, Cyrus Cooper, Stefania D’Angelo, Elizabeth M. Curtis, Sarah R. Crozier, Sheila J. Barton, Sian M. Robinson, Keith M. Godfrey, Nikki J. Graham, John W. Holloway, Nicholas J. Bishop, Stephen Kennedy, Aris T. Papageorghiou, Inez Schoenmakers, Robert Fraser, Saurabh V. Gandhi, Ann Prentice, Hazel M. Inskip, M. Kassim Javaid, the Maternal Vitamin D Osteoporosis Study Trial Group

**Affiliations:** 1 Medical Research Council Lifecourse Epidemiology Unit, University of Southampton, Southampton SO16 6YD, United Kingdom; 2 Paediatric Endocrinology, University Hospitals Southampton National Health Service Foundation Trust, Southampton SO16 6YD, United Kingdom; 3 National Institute for Health Research Southampton Nutrition Biomedical Research Centre, University of Southampton and University Hospital Southampton National Health Service Foundation Trust, Southampton SO16 6YD, United Kingdom; 4 National Institute for Health Research Musculoskeletal Biomedical Research Unit, University of Oxford, Oxford OX3 7LD, United Kingdom; 5 Human Development and Health, Faculty of Medicine, University of Southampton, Southampton SO16 6YD, United Kingdom; 6 Academic Unit of Child Health, Sheffield Children’s Hospital, University of Sheffield, Sheffield S10 2TH, United Kingdom; 7 Nuffield Department of Obstetrics and Gynaecology, John Radcliffe Hospital, University of Oxford, Oxford OX3 9DU, United Kingdom; 8 Medical Research Council Human Nutrition Research, Elsie Widdowson Laboratory, Cambridge CB1 9NL, United Kingdom; 9 Department of Medicine, Faculty of Medicine and Health Sciences, University of East Anglia, Norwich NR4 7TJ, United Kingdom; 10 Sheffield Hospitals National Health Service Trust (University of Sheffield), Sheffield S10 2SF, United Kingdom

## Abstract

**Context:**

Single-nucleotide polymorphisms (SNPs) in genes related to vitamin D metabolism have been associated with serum 25-hydroxyvitamin D [25(OH)D] concentration, but these relationships have not been examined following antenatal cholecalciferol supplementation.

**Objective:**

To determine whether SNPs in *DHCR7*, *CYP2R1*, *CYP24A1*, and *GC* are associated with the response to gestational cholecalciferol supplementation.

**Design:**

Within-randomization group analysis of the Maternal Vitamin D Osteoporosis Study trial of antenatal cholecalciferol supplementation.

**Setting:**

Hospital antenatal clinics.

**Participants:**

In total, 682 women of white ethnicity (351 placebo, 331 cholecalciferol) were included. SNPs at rs12785878 (*DHCR7*), rs10741657 (*CYP2R1*), rs6013897 (*CYP24A1*), and rs2282679 (*GC*) were genotyped.

**Interventions:**

1000 IU/d cholecalciferol from 14 weeks of gestation until delivery.

**Main Outcome Measure:**

25(OH)D at randomization and 34 weeks of gestation were measured in a single batch (Liaison; Diasorin, Dartford, UK). Associations between 25(OH)D and the SNPs were assessed by linear regression using an additive model [*β* represents the change in 25(OH)D per additional common allele].

**Results:**

Only rs12785878 (*DHCR7*) was associated with baseline 25(OH)D [*β* = 3.1 nmol/L; 95% confidence interval (CI), 1.0 to 5.2 nmol/L; *P* < 0.004]. In contrast, rs10741657 (*CYP2R1*) (*β* = −5.2 nmol/L; 95% CI, −8.2 to −2.2 nmol/L; *P* = 0.001) and rs2282679 (*GC*) (*β* = 4.2 nmol/L; 95% CI, 0.9 to 7.5 nmol/L; *P* = 0.01) were associated with achieved 25(OH)D status following supplementation, whereas rs12785878 and rs6013897 (*CYP24A1*) were not.

**Conclusions:**

Genetic variation in *DHCR7*, which encodes 7-dehyrocholesterol reductase in the epidermal vitamin D biosynthesis pathway, appears to modify baseline 25(OH)D. In contrast, the response to antenatal cholecalciferol supplementation was associated with SNPs in *CYP2R1*, which may alter 25-hydroxylase activity, and *GC*, which may affect vitamin D binding protein synthesis or metabolite affinity.

## Teaser

Associations between SNPs in the vitamin D metabolism pathway and 25(OH)D were assessed before and after antenatal vitamin D supplementation. The associations differed at the two time points.

Antenatal vitamin D supplementation is now recommended for all pregnant women in many national guidelines (1–3) as severe maternal vitamin D deficiency can result in symptomatic neonatal hypocalcemia (4). Furthermore, associations between maternal vitamin D status and obstetric complications (4) and offspring musculoskeletal development (5–7) have been reported.

Risk factors for vitamin D deficiency, in addition to geographic and seasonal variation, are well established. These include ethnicity, extent of skin covering, liberal use of sun protection, adiposity, and age. It is also increasingly recognized that genetic variation influences 25-hydroxyvitamin D [25(OH)D] status. In a previous genome-wide association study (GWAS), we demonstrated that a number of single-nucleotide polymorphisms (SNPs) in or near to genes encoding key components of the vitamin D metabolism pathway are associated with serum 25(OH)D level (8). These include *DHCR7* encoding 7-dehydrocholesterol (7-DHC) reductase; *CYP2R1* and *CYP24A1* encoding 25-hydroxylase and 24-hydroxylase, respectively; and *GC* encoding vitamin D binding protein (VDP). Several of these SNPs have been associated with the serum 25(OH)D increase in response to vitamin D supplementation in small studies (9–11).

Pregnancy is a physiologically unique period, involving hemodilution and hormonal and metabolic changes. For example, VDP rises early in pregnancy, and 1,25-dihydroxyvitamin D increases during the second and third trimesters (12). Supplementation with cholecalciferol increases maternal serum 25(OH)D concentration (7), and we have previously demonstrated that the 25(OH)D achieved in response to supplementation in pregnancy is associated with pregnancy weight gain, compliance, and baseline 25(OH)D (13). In the previous study, in contrast to data from nonpregnant adults, baseline body mass index (BMI), measures of adiposity, and maternal age were not associated with response to cholecalciferol supplementation in pregnancy (13). Considering this and the physiological changes to the vitamin D pathway in pregnancy, it is therefore possible that the genetic variants associated with baseline serum 25(OH)D status and the response to supplementation also differ between the pregnant and nonpregnant state. We therefore undertook this study to determine whether SNPs within the vitamin D metabolism pathway known to modify vitamin D status are also associated with the response to antenatal vitamin D supplementation.

## Materials and Methods

### The Maternal Vitamin D Osteoporosis Study

The Maternal Vitamin D Osteoporosis Study is a multicenter, double-blind, randomized, placebo-controlled trial of vitamin D supplementation in pregnancy. The primary outcome was neonatal bone mass. A detailed description of the study methods (14) and primary findings have been published previously (7). The study was approved by the Southampton and South West Hampshire Research Ethics Committee. The Maternal Vitamin D Osteoporosis Study was registered prospectively (ISRCTN:82927713; EUDRACT:2007-001716-23); full approval from UK Medicines and Healthcare Products Regulatory Agency was granted, and written, informed consent was obtained from all participants.

Briefly, women attending one of three UK hospitals [University Hospital Southampton National Health Service (NHS) Foundation Trust, Southampton, UK (latitude 50.9° North); Oxford University Hospitals NHS Foundation Trust, Oxford, UK (latitude 51.8° North); Sheffield Hospitals NHS Trust (University of Sheffield), Sheffield, UK (latitude 53.4° North)] for early pregnancy ultrasound screening (11 to 14 weeks of gestation) between 6 October 2008 and 11 February 2014 were invited to participate in the study. Inclusion criteria were as follows: age older than 18 years, singleton pregnancy, and gestation less than 17 weeks based on last menstrual period and ultrasound measurements. Women with known metabolic bone disease, renal stones, hyperparathyroidism or hypercalciuria, those taking medication known to interfere with fetal growth, fetal anomalies on ultrasonography, and women already using >400 IU/d vitamin D supplementation were excluded. A screening blood sample was obtained and analyzed on the local NHS platform [all three laboratories (Southampton, Oxford, and Sheffield) participate in the Vitamin D External Quality Assessment Scheme (http://www.deqas.org/)]. Women with 25(OH)D between 25 and 100 nmol/L and serum calcium <2.75 mmol/L were eligible to enroll fully in the study.

Participants were randomized to either cholecalciferol 1000 IU/d or matched placebo [Merck KGaA, Darmstadt, Germany/Sharp Clinical Services, Crickhowell, United Kingdom (previously DHP-Bilcare)], which was commenced before 17 weeks of gestation. Packs of study treatment were randomly assigned in a 1:1 ratio by Sharp Clinical Services by a computer-generated sequence in randomly permuted blocks of 10, starting randomly midway through the block, and sequentially numbered, before delivery to the study sites, and then dispensed in order by each study pharmacist. The study medication was provided in a blister pack in a single box containing all medication for the whole pregnancy. The participants, individuals providing antenatal and intrapartum care, and all field researchers involved in data collection and sample analysis were blinded to the assignment to the intervention. All participants received standard antenatal care and could continue self-administration of dietary supplements containing up to 400 IU/d vitamin D.

### Maternal assessments during pregnancy

Prior to commencing the study medication and again at 34 weeks of gestation, the women attended the research center for a detailed assessment of diet (including supplement use), lifestyle (smoking, physical activity participation, employment), and health (medical history, current medication use) using interviewer-led questionnaires. Ethnicity was reported by the participant and subsequently categorized as white or nonwhite.

Anthropometric measurements included height, measured to the nearest 0.1 cm using a stadiometer, and weight, assessed to the nearest 0.1 kg using calibrated electronic scales. Pregnancy weight gain was calculated as the difference between the weights at commencing the study medication and at 34 weeks of gestation.

Compliance with study medication was assessed by asking participants to bring any remaining study medication to each assessment. The pills were counted and compliance calculated as the number consumed divided by the expected consumption based on the number of days since the medication was dispensed and expressed as a percentage.

### Assessment of 25(OH)D

On the day that the study medication was dispensed and at 34 weeks of gestation, a nonfasted venous blood sample was obtained and serum stored at −80°C. 25(OH)D concentration was assessed by chemiluminescence immunoassay (Liaison automated platform; Diasorin, Dartford, UK). All samples were analyzed in a single batch at the end of the study at Medical Research Council Human Nutrition Research (Cambridge, United Kingdom). Details of assay performance and quality control through participation in Vitamin D External Quality Assessment Scheme, National Institute of Standards and Technology, and UK National External Quality Assessment Service are given elsewhere (15, 16).

### Analysis of SNPs

Genotyping was undertaken by LGC Genomics (Hoddeston, UK) using KASP competitive allele-specific polymerase chain reaction. SNPs selected for analysis were based on the findings of a previous GWAS (8). These were rs12785878 (*DHCR7*), rs10741657 (*CYP2R1*), rs6013897 (*CYP24A1*), and rs2282679 (*GC*).

### Statistical analysis

Women who had a measurement of 25(OH)D at both 14 and 34 weeks of gestation, had genetic analysis, and delivered a live-born infant were included in the analysis. The SNPs included in this analysis were chosen based on findings in the previous GWAS, which included only individuals of European ancestry (8). We therefore limited our study population to only women of white ethnicity (95.8% of eligible women).

All outcomes were assessed for normality using visual inspection. Serum 25(OH)D concentrations at 14 and 34 weeks of gestation were normally distributed. Characteristics of the women in the two treatment arms were compared using the *t* test, Mann-Whitney *U* test, and *χ*^2^ test for normally distributed, nonnormally distributed, and categorical variables, respectively. All participants were analyzed in the group to which they were originally randomized.

Linear regression was used to examine the association between the four SNPs and the outcomes using an additive model with the homozygous low-frequency allele (for this cohort) as the reference group. The additive model thus expresses the change in outcome per additional common allele. The outcomes assessed were 25(OH)D at 14 weeks of gestation in all study participants and the achieved 25(OH)D at 34 weeks of gestation in the participants randomized to cholecalciferol. In addition, in women randomized to cholecalciferol, we assessed the relationship between the SNPs and the change in vitamin D using the residuals of 25(OH)D at 34 weeks of gestation regressed on 25(OH)D at 14 weeks of gestation as the outcome. Multivariate linear regression was then used to adjust for relevant confounders. Maternal age, BMI, physical activity and smoking status at 14 weeks of gestation, educational attainment, and season of blood measurement were included in the model for baseline 25(OH)D as these have been shown to be associated with serum 25(OH)D status in pregnancy (17–21). For the models for the 25(OH)D achieved at 34 weeks of gestation, compliance with study medication, baseline 25(OH)D, and pregnancy weight gain (instead of BMI) were also included as we have previously demonstrated in this cohort of women that these factors are associated with the response to cholecalciferol supplementation (13). Seasons were defined according to the UK Meteorological Office recommendations (www.metoffice.gov.uk) with winter (December to February), spring (March to May), summer (June to August), and autumn (September to November). As four SNPs were assessed, a Bonferroni correction was used to account for multiple testing. We also undertook sensitivity analysis in which women who reported having taken any additional vitamin D–containing supplements within 90 days of the late-pregnancy blood sampling were excluded.

Finally, to determine the combined effects of SNPs, a genotype risk score (GRS) was calculated as the sum of the number of risk alleles at rs10741657 (G) and rs2282679 (C), as identified in the previous analysis. The GRS score ranged from 0 to 4, with a score of 0 representing no risk alleles. Multivariate linear regression was used to determine the association of GRS with 25(OH)D, using the same confounding factors as before.

All analyses were performed in Stata v14.2 (StataCorp, College Station, TX). A *P* value of <0.05 was considered statistically significant.

## Results

**Figure 1.**
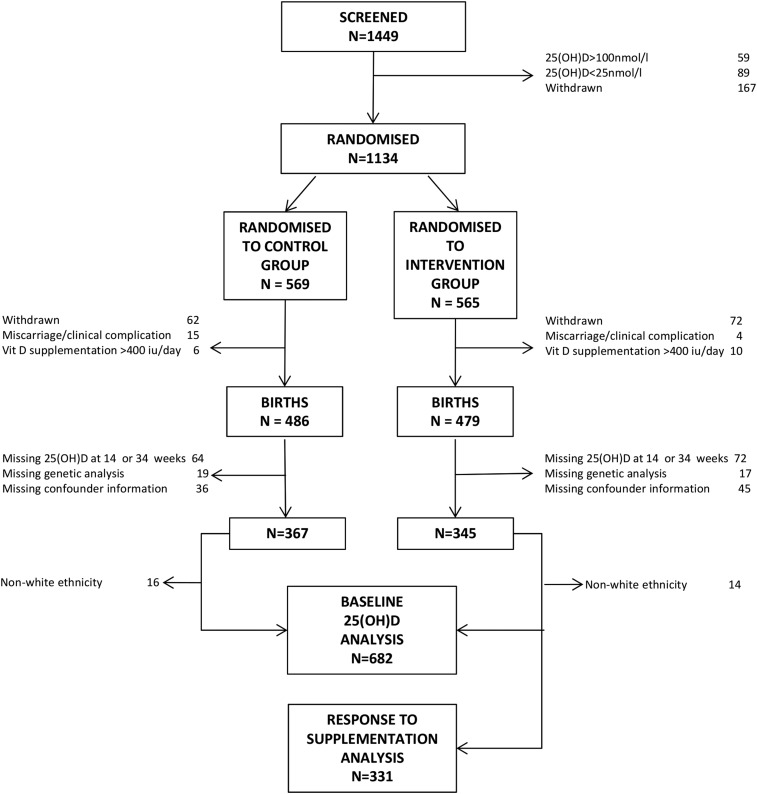
Consolidated Standards of Reporting Trials diagram.

In total, 682 women were included in the analysis (Fig. 1). Women who delivered a live-born infant but who were not included in this analysis due to missing 25(OH)D or genetic analysis were of similar age, smoking status, and BMI to those included in the analysis (*P* > 0.05 for all). Baseline characteristics of the women randomized to placebo and cholecalciferol were similar (Table 1). The distributions of alleles within the SNPs of interest were also similar between the two groups (Table 1).

**Table 1.** **Characteristics of Women Included in the Analysis**

Characteristic	**Placebo (n = 351)**	**Cholecalciferol 1000 IU/d (n = 331)**
Age (years), mean (SD)	30.8 (5.4)	30.8 (5.0)
Current smoking, n (%)	29 (8.3)	24 (7.3)
Educational attainment ≥ A level (high school), n (%)	258 (74.4)	264 (80.0)
BMI (kg/m^2^), median (IQR)	25.5 (23.0 to 29.7)	24.7 (22.3 to 29.3)
Pregnancy weight gain (kg), mean (SD)	9.5 (3.6)	9.5 (3.4)
Strenuous exercise ≥ once per week, n (%)	45 (13.8)	50 (16.2)
25(OH)D at 14 weeks (nmol/L), mean (SD)	45.4 (16.5)	46.1 (17.0)
rs12785878 (*DHCR7*), n (%)		
G:G	17 (4.8)	14 (4.3)
T:G	114 (32.5)	120 (36.6)
T:T	220 (62.7)	194 (59.1)
rs10741657 (*CYP2R1*), n (%)		
A:A	57 (16.6)	51 (15.5)
G:A	156 (45.3)	148 (45.1)
G:G	131 (38.1)	129 (39.3)
rs6013897 (*CYP24A1*), n (%)		
A:A	10 (2.9)	15 (4.6)
T:A	126 (36.5)	102 (31.4)
T:T	209 (60.6)	208 (64.0)
rs2282679 (*GC*), n (%)		
C:C	37 (10.6)	34 (10.3)
C:A	149 (42.6)	136 (41.3)
A:A	164 (46.9)	159 (48.3)

Abbreviation: IQR, interquartile range.

25(OH)D was similar at baseline between the two groups but significantly higher in the women randomized to cholecalciferol at 34 weeks of gestation [mean (standard deviation), 67.3 (20.8) nmol/L] compared with placebo [mean (standard deviation), 43.0 (22.2) nmol/L, *P* < 0.001].

### Associations with baseline 25(OH)D

Among all 682 women, the common allele (T) of rs12785878 (*DHCR7*) was associated with greater baseline serum 25(OH)D concentration (Table 2). This association persisted in multivariate analysis adjusting for maternal age, BMI, smoking status, physical activity, educational achievement and season of blood sampling [*β* = 3.1 nmol/L per T allele; 95% confidence interval (CI), 1.0 to 5.2 nmol/L; *P* = 0.016]. There were no statistically significant associations between the SNPs at rs10741657 (*CYP2R1*), rs6013897 (*CYP24A1*), and rs2282679 (*GC*) and baseline 25(OH)D in the fully adjusted model (Table 2).

**Table 2.** **Association of SNPs With Baseline 25(OH)D in Early Pregnancy**

**SNP**	**Reference Allele**	**Common Allele**	**Univariate**			**Adjusted*****^a^***			**Corrected *P* Value*****^b^***
			**n**	***β* (95% CI)**	***P* Value**	**n**	***β* (95% CI)**	***P* Value**	
rs12785878 (*DHCR7*)	G	T	679	3.7 (1.5 to 5.8)	0.001	622	3.1 (1.0 to 5.2)	0.004	0.012
rs10741657 (*CYP2R1*)	A	G	672	−1.1 (−2.9 to 0.7)	0.24	616	−1.4 (−3.2 to 0.4)	0.12	0.47
rs6013897 (*CYP24A1*)	A	T	670	1.2 (−1.1 to 3.4)	0.31	613	0.8 (−1.4 to 3.0)	0.46	1.0
rs2282679 (*GC*)	C	A	679	2.2 (0.3 to 4.1)	0.02	622	1.7 (−0.2 to 3.5)	0.08	0.31

The homozygous low-frequency gene variant was used as the reference group, and *β* represents the change in 25(OH)D (nmol/L) per common allele.

^a^ Adjusted for age, BMI, smoking status (yes/no), physical activity (strenuous activity more than once per week, yes/no), educational achievement (A levels or higher, yes/no), and season of blood sampling (winter, spring, summer, autumn).

^b^ Bonferroni-corrected *P* values.

### Associations with 25(OH)D following supplementation

**Figure 2.**
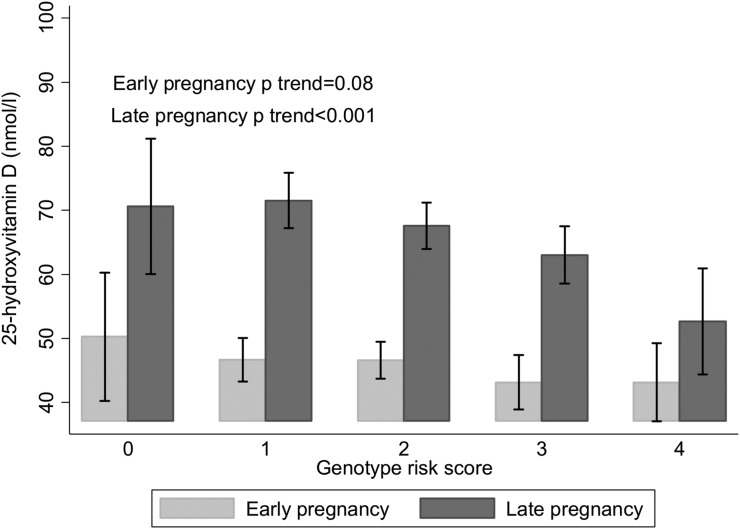
25(OH)D before and after supplementation with cholecalciferol in pregnancy according to GRS for the SNPs rs10741657 (*CYP2R1*) and rs2282679 (*GC*). Shown as mean and 95% CI for each group. *P* is for trend by linear regression with adjustment for confounders.

In women who were randomized to cholecalciferol supplements, the common G allele of rs10741657 (*CYP2R1*) was associated with lower serum 25(OH)D concentration at 34 weeks of gestation, whereas the common A allele of rs2282679 (*GC*) was associated with greater 34-week serum 25(OH)D (Table 3). These associations persisted after adjustment for potential confounding factors (maternal age, physical activity, and smoking status at 14 weeks of gestation; educational attainment; season of blood measurement; compliance with study medication; baseline 25(OH)D; and pregnancy weight gain). There were no significant associations between the SNPs at rs12785878 (*DHCR7*) and rs6013897 (*CYP24A1*) and 25(OH)D at 34 weeks of gestation (Table 3). When both rs10741657 (*CYP2R1*) (*β* = −5.0 nmol/L per G allele; 95% CI, −8.0 to −1.9 nmol/L per G allele; *P* = 0.001) and rs2282679 (*GC*) (*β* = 3.8 nmol/L per A allele; 95% CI, 0.6 to 7.1 nmol/L per A allele; *P* = 0.021) were included simultaneously in a regression model with confounders, the effect sizes were maintained. Furthermore, a GRS including rs10741657 and rs2282679 showed that for each additional risk allele, late pregnancy serum 25(OH)D concentration after supplementation with cholecalciferol was lower by 4.4 nmol/L (95% CI, 2.3 to 6.6 nmol/L, *P* < 0.001). This association between the GRS and 25(OH)D was not present presupplementation in early pregnancy (Fig. 2).

**Table 3.** **Association of SNPs With Achieved 25(OH)D at 34 Weeks of Gestation Following Supplementation With 1000 IU/d Cholecalciferol in Pregnancy**

**SNP**	**Reference Allele**	**Common Allele**	**Univariate**			**Adjusted*****^a^***			**Corrected *P* Value*****^b^***
			**n**	***β* (95% CI)**	***P* Value**	**n**	***β* (95% CI)**	***P* Value**	
rs12785878 (*DHCR7*)	G	T	328	2.6 (−1.4 to 6.5)	0.20	304	0.3 (−3.5 to 4.0)	0.89	1.0
rs10741657 (*CYP2R1*)	A	G	328	−4.8 (−8.0 to −1.6)	0.004	304	−5.2 (−8.2 to −2.2)	0.001	0.004
rs6013897 (*CYP24A1*)	A	T	325	−3.2 (−7.1 to 0.8)	0.11	301	−1.0 (−4.8 to 2.8)	0.60	1.0
rs2282679 (*GC*)	C	A	329	4.3 (0.9 to 7.7)	0.01	305	4.2 (0.9 to 7.5)	0.01	0.04

The homozygous low frequency gene variant was used as the reference group. *β* represents the effect on achieved 25(OH)D (nmol/L) per common allele.

^a^ Adjusted for age, pregnancy weight gain, smoking status (yes/no), physical activity (strenuous activity more than once per week, yes/no), educational achievement (A levels or higher, yes/no), season of blood sampling (winter, spring, summer, autumn), compliance with study protocol, and baseline 25(OH)D.

^b^ Bonferroni-corrected *P* values.

**Figure 3.**
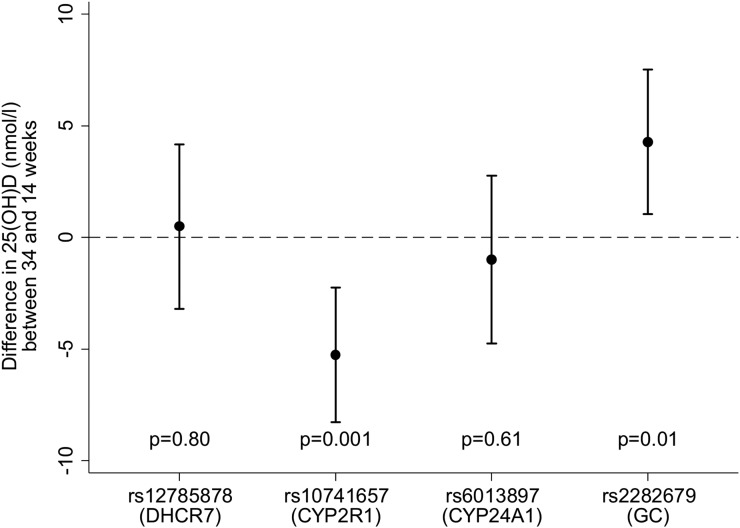
Associations between SNPs and change in 25(OH)D from 14 to 34 weeks of gestation following supplementation with 1000 IU/d cholecalciferol. Shown as *β* and 95% CI. The homozygous low-frequency allele was used as the reference group, with the *β* representing the change in 25(OH)D (nmol/L) per common allele (additive model). Models were adjusted for age, physical activity, smoking status, educational attainment, season of blood sampling, compliance, and pregnancy weight gain.

Similarly, rs10741657 (*CYP2R1*) and rs2282679 (*GC*) were associated with the change in 25(OH)D from 14 to 34 weeks of gestation in women who received the cholecalciferol supplement, whereas rs12785878 (*DHCR7*) and rs6013897 (*CYP24A1*) were not (Fig. 3). The GRS was also negatively associated with the change in serum 25(OH)D concentration (*β* = −4.4 nmol/L per risk allele; 95% CI, −6.6 to −2.3 nmol/L per risk allele; *P* < 0.001), such that women who had more risk alleles had a smaller increment in 25(OH)D.

### Sensitivity analysis

As the women were allowed to continue taking vitamin D supplements containing up to 400 IU/d, in sensitivity analyses, we included only women who were not taking additional vitamin D–containing supplements at 34 weeks of gestation (n = 224, 67.7%). The relationships were similar to those in the whole cohort.

## Discussion

This study assessed the relationships between common genetic variants and the response to vitamin D supplementation in pregnancy; rs12785878 in *DHCR7* was associated with 25(OH)D status in early pregnancy prior to vitamin D supplementation, whereas rs10741657 in *CYP2R1* and rs2282679 in *GC* were associated with both the achieved and change in 25(OH)D concentration following supplementation with 1000 IU/d cholecalciferol.

GWAS has been used to identify SNPs associated with serum 25(OH)D status (8, 22). However, there are few studies investigating whether common genetic variants modify the response to supplementation and, to our knowledge, no previous studies in pregnant women. Two previous studies in nonpregnant adults similarly found that rs2282679 (*GC*) and rs10741567 (*CYP2R1*) are associated with serum 25(OH)D increment in response to supplementation (10, 11), whereas Barry *et al.* (9) did not identify these associations. The observed difference in achieved 25(OH)D between the homozygous gene variants was higher in the studies by Sollid *et al.* (10) and Didriksen *et al.* (11) (15 to 18 nmol/L) than our cohort (9 to 10 nmol/L), but this could reflect the higher cholecalciferol doses used in those studies and/or alterations in vitamin D metabolism and volume of distribution in pregnancy.

In our population, the SNPs associated with baseline 25(OH)D differed from those associated with the response to cholecalciferol supplementation. The published GWAS (8, 22) did not stratify individuals by vitamin D supplement usage, which could account for differing associations before and after supplementation in our cohort, or the differing findings might reflect the lower power in our study. Nonetheless, although the functional consequences of these genetic markers remain the subject of research, these differences are biologically plausible. The *DHCR7* gene encodes 7-DHC reductase, which converts 7-DHC back to cholesterol, thereby reducing the availability of 7-DHC for conversion to previtamin D. The association of this SNP with only baseline 25(OH)D is biologically consistent with the notion that the relative proportion of 25(OH)D obtained from vitamin D biosynthesis in the skin as opposed to dietary intake is lower following supplementation.

*CYP2R1* encodes a 25-hydroxylase, a key enzyme involved in the conversion of vitamin D to 25(OH)D (23). Hepatic hydroxylation of previtamin D to 25(OH)D is thought not to be regulated and is therefore primarily dependent on availability of the substrate. We found that the rs10741657 SNP was not associated with baseline 25(OH)D but was associated with the response to gestational vitamin D supplementation, therefore suggesting that this SNP only modifies 25(OH)D status when the substrate is more readily available and that at the baseline measurement, saturation of the enzyme had not been reached. rs10741657 is located within the promoter region of the *CYP2R1* gene, and our findings would suggest that presence of the A allele increases enzyme production. Because the G allele at rs10741657, which was more frequent in our population, was associated with lower 25(OH)D following supplementation, it could be postulated that this allele previously conferred an evolutionary advantage to prevent vitamin D toxicity.

In addition to associations with 25(OH)D, rs2282679 in *GC* has also been associated with serum VDP concentrations, with carriers of the low-frequency C allele having reduced concentrations of VDP and 25(OH)D (8). SNPs in *GC* have also been associated with the binding affinities of 25(OH)D to VDP (24), although the effect of rs2282679 on binding affinity has not been established. We similarly found that the C allele was associated with lower achieved 25(OH)D following antenatal vitamin D supplementation. Unfortunately, analysis of VDP was not available in this cohort of women.

It is well recognized that individuals with darker skin pigmentation living at higher latitudes tend to have lower 25(OH)D. This may in part reflect clustering of genotypes within ethnic groups. For example, the G allele at rs12785878 (*DHCR7*) is typically more prevalent in nonwhite populations (25–29). Although our study included only women of white ethnicity, the G allele was associated with lower baseline 25(OH)D. The greater prevalence of the G allele at rs12785878 with a resulting increase in 7-DHC reductase activity (either due to a functional modification or increased synthesis) leading to reduced availability of 7-DHC for conversion to previtamin D might contribute to lower 25(OH)D in individuals with darker skin pigmentation. Furthermore, it has previously been shown that rs2282679 (*GC*) was only significantly associated with 25(OH)D in European Americans and not African Americans (30), suggesting further ethnicity-specific associations, which might form the basis of future study, and consideration in clinical approaches to supplementation.

There are a number of limitations to our findings. First, due to stipulations made during the ethics approval process, we were unable to include participants with a baseline 25(OH)D less than 25 nmol/L or greater than 100 nmol/L. As such, confirmation of these findings in severely vitamin D–deficient women is needed as it is possible that women with specific genotypes were selectively excluded by this inclusion criterion. Second, we did not identify associations between several SNPs and either baseline and/or achieved 25(OH)D that had previously been associated with 25(OH)D in GWAS (8, 22). This may reflect the lower power of this study, although our findings are similar to other small studies (10, 11). Furthermore, in this study, we performed analysis only of candidate SNPs previously identified to be associated with differences in 25(OH)D status. However, it is possible that alternate SNPs/genes that are not clinically important to 25(OH)D level in nonpregnant adults would be significant in pregnant women and could be identified by GWAS.

In conclusion, common genetic variation is associated with baseline 25(OH)D in pregnancy and the response to antenatal supplementation with 1000 IU/d cholecalciferol, but with differing SNPs appearing to be important before and after supplementation. Our findings suggest that analysis of SNPs may have an important role in identifying high-risk categories of individuals who are likely to require higher doses of vitamin D to achieve repletion, and studies are needed to establish clinical approaches to vitamin D supplementation that are centered on individual characteristics.
